# Long-Term Aging Behavior of Plastic/Styrene Butadiene Rubber (SBR) Composite Modified Bitumen

**DOI:** 10.3390/ma16134567

**Published:** 2023-06-24

**Authors:** Chengwei Xing, Mingchen Li, Lingxiao Liu, Ruikang Yang

**Affiliations:** 1 Key Laboratory for Special Area Highway Engineering of Ministry of Education, Chang’an University, South 2nd Ring Road Middle Section, Xi’an 710064, China; xingcw@chd.edu.cn; 2 School of Highway, Chang’an University, South 2nd Ring Road Middle Section, Xi’an 710064, China; 3 The Key Laboratory of Road and Traffic Engineering, Ministry of Education, Tongji University, Shanghai 201800, China; 4 China State Construction Engineering (Hong Kong) Limited, Hong Kong, China; lingxiao.liu@cohl.com

**Keywords:** waste plastic, SBR, long-term aging behavior, rheological properties, molecular weight distribution

## Abstract

The reuse of recycled waste plastics has long been attempted in pavement engineering as bitumen modifier. It was revealed that waste plastics can significantly enhance the high-temperature performance of bitumen and bitumen mixtures. Even so, the application of waste plastics as a bitumen modifier is still not widespread. This is attributable to the generally poor low-temperature performance of plastic-modified bitumen, which often fails to meet specification requirements. For this purpose, styrene butadiene rubber (SBR) was selected to improve the low-temperature performance of plastic-modified bitumen. However, due to the long-term aging process, the composite and structure of the modified bitumen will change, which negatively impacts its performance. The objective of this study is to investigate the long-term aging behavior of plastic/SBR composite-modified bitumen. For this purpose, waste polyethylene was used as a plastic modifier and was mixed with base bitumen and 3% SBR at ratios 4.5%, 6% and 7.5%. The rheological properties and molecular weight distribution of base bitumen, plastic and plastic/SBR-modified bitumen before and after long-term aging were measured. Results show that the incorporation of plastic can improve the complex modulus, rutting factor and percent recovery of bitumen and reduce the non-recoverable creep compliance of the bitumen, indicating the modification process enhances the high-temperature performance of bitumen. The enhancement effect is more pronounced with the increase of plastic content. For modified bitumen with 7.5% plastic modifier, the complex modulus of modified bitumen is increased by 1127.55% compared to base bitumen. The addition of 3% SBR modifier can further improve the high-temperature performance of the modified bitumen. In addition, the modification process also increases the large molecule size percentage (LMSP) and weight average molecular weight of bitumen. Compared with weight average molecular weight, the LMSP correlates well with the rheological properties of modified bitumen. In accordance with the complex modulus, using the LMSP and weight average molecular weight of bitumen before and after aging, the corresponding aging index was calculated. The quantitative results showed that the addition of plastic modifier can improve the aging resistance of bitumen, but the enhancement effect is not as obvious as that of SBR modifier.

## 1. Introduction

In recent years, the recycling of waste materials has received increasing attention [1,2,3,4]. As the consumption of plastic products has increased, the amount of waste plastic has also increased [5,6,7]. It is estimated that the global production of waste plastics amounts to 300 million tons, but less than 20% of waste plastics are recycled. Most waste plastics are landfilled or incinerated, which causes serious environmental pollution problems. In recent years, there have been many attempts to recycle waste materials in the field of pavement engineering, and the reuse of waste plastics as modifiers for bitumen has become a major application trend [8,9,10]. Related studies have revealed that waste plastics can significantly enhance the high-temperature performance of bitumen and its mixtures. For instance, with the aid of the multiple stress creep recovery (MSCR) test, Al-Abdul et al. [11] investigated the effects of three kinds of waste plastics modifiers (recycled polypropylene and low- and high-density polyethylene) on the high-temperature performance of composite-modified bitumen. Their findings have shown that the addition of waste plastics can affect the percentage recovery and non-recoverable creep of bitumen and hence significantly improve the high performance of bitumen [11].

Nevertheless, waste plastic-modified bitumen is still not extensively used in practical engineering. There are various reasons why waste plastic-modified bitumen is not yet widely used. Firstly, there are many different types of waste plastics, and their chemical composition varies, which leads to large differences in performance between different forms of plastic-modified bitumen and its mixtures. Secondly, due to the high modulus of waste plastic-modified bitumen, pavement made from it is prone to cracking at low temperatures [12,13]. In particular, because the low temperature ductility of plastic-modified bitumen struggles to meet the requirements of the relevant specifications, it greatly limits the widespread use of plastic-modified bitumen. For this reason, the incorporation of rubber materials such as crumb rubber, styrene-butadiene-styrene (SBS) and styrene butadiene rubber (SBR) is commonly adopted to improve the low-temperature performance of waste plastic-modified bitumen [14,15,16]. Considering that composite modification using SBR can change the chain rigidity and crosslink density of composite materials, SBR is an effective modifier commonly used to enhance the low-temperature performance of modified bitumen in practical engineering [17,18]. Ren et al. [18] investigated the effect of the addition of the SBR modifier on the low-temperature crack resistance of gilsonite-modified bitumen using the blending beam rheometer (BBR) test and found that the addition of SBR is a valuable way to improve the low-temperature PG grade of gilsonite-modified bitumen. Liu et al. [19] used SBR modifier to enhance the low-temperature performance of Buton rock-modified bitumen mixtures and revealed that the bending strength and maximum bending strain of SBR/Buton rock composite-modified bitumen mixtures at −10 °C were increased by 10% and 65%, respectively, following the addition of SBR. As such, using SBR to modify bitumen is an effective solution to enhance the low-temperature performance of waste plastic-modified bitumen.

In addition to low-temperature performance, the effect of aging on waste plastic-modified bitumen is also of concern. During the service life of bitumen pavement, bitumen is subject to aging due to multiple environmental factors [20,21,22,23,24]. After aging, bitumen becomes hardened and brittle, while the chemical composition of the bitumen also changes significantly [25,26,27]. It cracks and breaks easily under the action of external forces and fails to continue to perform its original role of bonding. Therefore, the aging behavior of modified bitumen has been a focus of interest for researchers. By comparing the rheological and chemical properties of SBS-modified bitumen, Lin et al. [28] suggested that the addition of a modifier can retard the aging of bitumen. However, most of the existing research on the long-term aging behavior of bitumen has focused on the more commonly used types of modified bitumen, such as SBS-modified bitumen. There are relatively few studies on the long-term aging behavior of plastic-modified bitumen. In addition, the aging behavior of composite-modified bitumen is more complicated than that of single-modified bitumen. Wang et al. [29] investigated the evolution of the rheological and chemical properties of SBS-modified bitumen and terminal blend rubberized/SBS composite-modified bitumen due to long-term aging and pointed out that the incorporation of terminal blend rubberized bitumen slows the aging rate of SBS-modified bitumen. As such, it is useful to investigate the long-term aging behavior of plastic/SBR composite-modified bitumen.

The objective of this study is to analyze the long-term aging behavior of plastic/SBR composite-modified bitumen. For this purpose, the waste plastic and SBR modifiers were first mixed with base bitumen to prepare the composite-modified bitumen. Following this, the prepared modified bitumen was subject to a pressure aging vessel (PAV) test to simulate long-term aging during service life. Then, temperature sweep and multiple stress creep recovery tests of the virgin and long-term aged modified bitumen were conducted to evaluate the evolution of the rheological properties of bitumen. At the same time, the evolution of the chemical composition of prepared bitumen was tested with the aid of gel permeation chromatography (GPC). Finally, the correlation between the rheological properties and chemical properties of waste plastic/SBR composite-modified bitumen was analyzed.

## 2. Materials and Methods

First, a brief description of the materials and methods used in this study is given. Meanwhile, to facilitate the reader’s understanding, Table 1 summarizes the abbreviations used in this studies.

### 2.1. Materials

In this study, the plastic modifier selected is a common household waste polyethylene material. The waste plastic modifier is mixed with a kind of PG 64-22 base bitumen to prepare the plastic-modified bitumen. The contents of waste plastic in the modified bitumen are 4.5%, 6% and 7.5% by weight. In addition, a kind of commercial SBR latex provided by a local company is used as modifier to enhance the low-temperature performance of plastic-modified bitumen. The Mooney viscosity of latex is 4000 MPa·s. The content of SBR modifier in the composite-modified bitumen is 3% by weight, Table 2 shows the basic properties of bitumen used in this study.

### 2.2. Methods

#### 2.2.1. Preparation Process of Composite Modified Bitumen

The detailed preparation process for modified bitumen is as follows: First, the base bitumen is heated to 160 °C, and the weighed waste plastic modifier (4.5%, 6% and 7.5% by weight) is added to the heated bitumen. At this temperature, the plastic is left to swell for approximately 45 min. During the swelling process, a glass rod is used for stirring to disperse the plastic modifier into small pieces. Following this, the swollen bitumen is subjected to shearing using a high-speed shear at 170 °C for 30 min; the shear speed is set as 5000 r/min. For plastic/SBR-modified composite bitumen, the weighed SBR (3% by weight) is then added into the plastic bitumen for another 30 min high-speed shear process. The shear temperature is set as 170 °C and the shear speed is set as 5000 r/min. Finally, the mixed modified bitumen is stored at ambient temperature in preparation for testing.

#### 2.2.2. Aging Methods

The long-term aging of base and modified bitumen is simulated via PAV test according to AASHTO R28. The parameters in the PAV test are set as follows: the aging temperature is controlled as 100 °C and the aging period is set as 20 h. This aging condition is regarded as simulating the aging process of bitumen pavements over a period of 5 to 10 years. Before PAV test, an 85-min rolling thin film oven test was conducted at 163 °C for base and modified bitumen according to AASHTO T240 to simulate the short-term aging of bitumen during mixing and paving. Table 3 presents the detailed preparation process of bitumen used in this study and their identifications.

#### 2.2.3. Temperature Sweep (TS) Test

The complex modulus (G*) and phase angle (δ) of base bitumen, plastic-modified bitumen and plastic/SBR composite-modified bitumen before and after long-term aging at 58 °C, 64 °C, 70 °C, 76 °C and 82 °C, respectively, were measured via TS test. To compare the variation patterns of the complex modulus and phase angle of the bitumen samples under different aging conditions, the same test parameters were used for all samples. The bitumen samples were loaded in controlled-strain mode with a 10 rad/s ± 0.1 rad/s sinusoidal oscillation load. The strain levels are all 10%.

#### 2.2.4. Multiple Stress Creep Recovery (MSCR) Test

The MSCR test is an improved test method based on the repeated creep recovery test, which is primarily used to evaluate the high-temperature performance and elastic response of bitumen materials. Based on ASSHTO TP-350, MSCR tests were performed on the base bitumen, plastic-modified bitumen and plastic/SBR composite-modified bitumen before and after long-term aging at 58 °C, 64 °C, 70 °C, 76 °C and 82 °C. In each MSCR test, twenty creep-and-recovery cycles at 0.1 kPa stress were first conducted on the prepared bitumen sample, followed by ten creep-and-recovery cycles at 3.2 kPa stress. Each creep-and-recovery process consists of a 1 s creep process and a 9 s recovery process. The time-strain data during the creep-and-recovery process was automatically collected by the dynamic shear rheometer. Based on the collected data, the following indicators were calculated.

(1) $$R=\frac{\varepsilon_{p}-\varepsilon_{u}}{\varepsilon_{p}}\times 100\%$$

(2) $$J_{nr}=\frac{\varepsilon_{u}}{\sigma }$$

where *R* presents the percent recovery, %; *J_nr_* represents non-recoverable creep compliance, kPa^−1^; $\varepsilon_{p}$ presents peak strain, %; $\varepsilon_{u}$ presents unrecovered strain, %; σ refers to the applied shear stress in kPa.

#### 2.2.5. GPC Test

The GPC is an effective instrument for separating substances to be measured according to their molecular weight. During the GPC test, the sample solution flows slowly across the column containing the porous gel. The smaller the molecules, the simpler it is for molecules to enter the micro-pores inside the gel. Thus, smaller molecules pass through the column more slowly and are retained in the column longer. In contrast, the larger molecules have difficulty entering the microporous pores and are therefore the first to exit the column and be detected. The retention times in the chromatograms range from short to long, corresponding to the size of the molecules from large to small.

In this study, the GPC test was performed on base bitumen, plastic-modified bitumen and plastic/SBR composite-modified bitumen to evaluate the evolution of the molecular weight distribution of bitumen due to long-term aging. To prepare the bitumen sample for the GPC test, the selected samples were first dissolved in tetrahydrofuran solution; the concentration of the solutions was all controlled to 3.2 mg/mL. The bitumen and tetrahydrofuran solution was soaked for one day and then filtered through a 0.45 μm sieve. The filtered sample was then injected into the GPC instrument to obtain its molecular weight distribution chromatogram.

## 3. Results and Discussion

### 3.1. Evaluation of Complex Modulus G*

**Figure 1:**
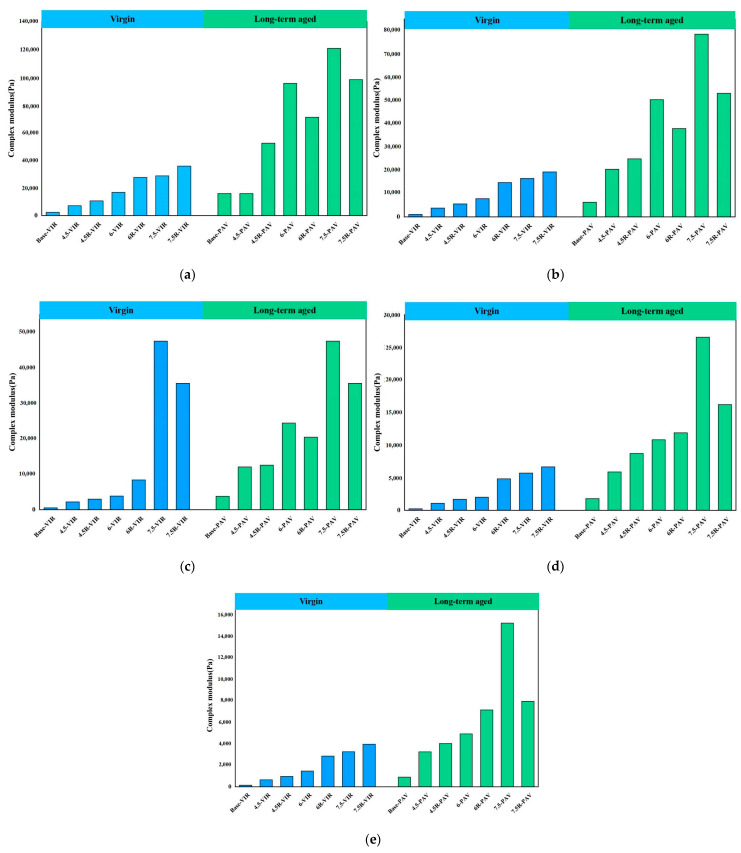
Complex modulus of bitumen before and after aging at: (**a**) 58 °C; (**b**) 64 °C; (**c**) 70 °C; (**d**) 76 °C; (**e**) 82 °C.

Figure 1 presents the complex modulus G* of different bitumen samples before and after long-term aging at 58 °C, 64 °C, 70 °C, 76 °C and 82 °C, respectively. It can be seen from the figure that as the temperature increases, the complex modulus of bitumen sample tends to decrease. The development of complex modulus is attributed to the increase in temperature intensifying the irregular movement of bitumen molecules. As such, less stress is required for the same strain response, reducing the resistance of bitumen to external forces, which finally expresses a reduction in the complex modulus. In addition, the addition of waste plastic increases the complex modulus of the base bitumen. Using 58 °C as an example, the addition of 4.5%, 6% and 7.5% plastic modifier to the base bitumen increases the complex modulus by 205.26%, 619.63% and 1127.55%, respectively. The increase in complex modulus is more pronounced with increasing amounts of plastic incorporated. The incorporation of SBR modifier does not only increase the complex modulus of plastic-modified bitumen. The above results suggest that the integrated modification enhances the permanent deformation resistance of bitumen.

After long-term aging, the complex modulus of different modified bitumen increases substantially. In existing studies, the resistance of bitumen to aging is usually evaluated via complex modulus aging index (CAI), which is the ratio of complex modulus of bitumen after aging to that of bitumen before aging. In this study, to compare the aging resistance of different bitumen samples, the CAI of base bitumen, plastic-modified bitumen and plastic/SBR composite-modified bitumen were calculated; results are shown in Table 4. As can be seen from the table, the CAI of base bitumen is the highest, indicating that after long-term aging, the complex modulus of base bitumen varies most significantly. In contrast, the aging index CAI of bitumen gradually decreases with the incorporation of plastic modifier. Using 58 °C as an example, the addition of 4.5%, 6% and 7.5% plastic modifier to the base bitumen decreases the CAI by 12.7%, 16.7% and 38.3%, respectively. The decrease in CAI becomes more pronounced as the amount of plastic modifier is increased. This indicates that the addition of plastic modifier has improved the aging resistance of bitumen. In addition, the integrate modification of plastic and SBR resulted in the smallest CAI for composite-modified bitumen. The CAI of SBR/plastic composite-modified bitumen is far less than that of plastic-modified bitumen, indicating that compared with plastic, the application of SBR is more effective in improving the aging resistance of bitumen. For plastic/SBR composite-modified bitumen with 6% and 7.5% plastic inclusion, the aging index CAI is only around 2.5. As such, 6% and 7.5% plastic inclusion are recommended to produce plastic/SBR composite-modified bitumen to obtain the bitumen with excellent aging resistance.

### 3.2. Evaluation of Phase Angle δ

**Figure 2:**
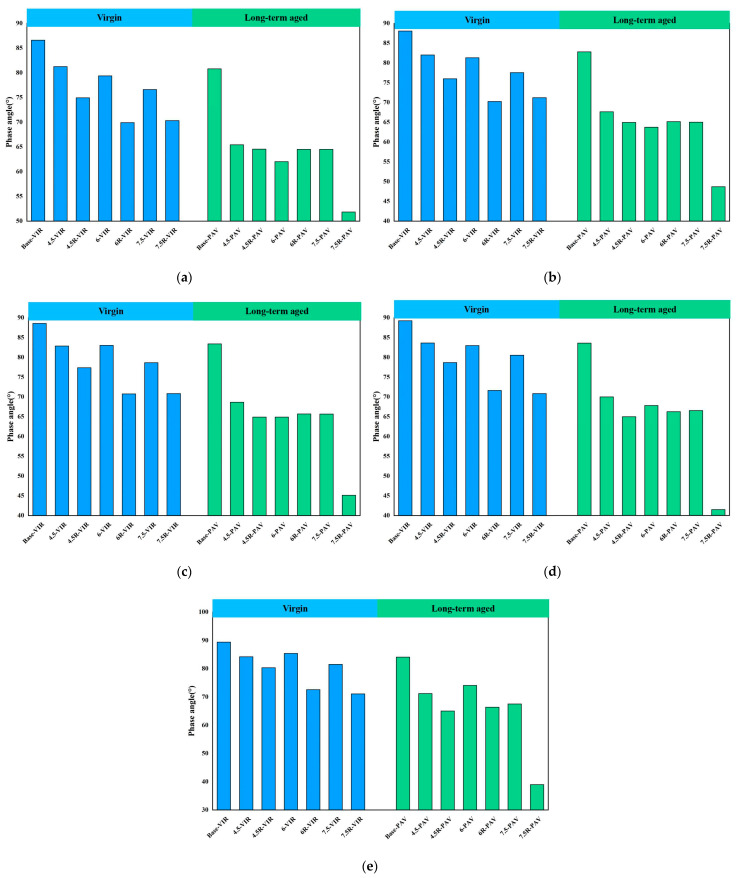
Phase angle of bitumen before and after aging at: (**a**) 58 °C; (**b**) 64 °C; (**c**) 70 °C; (**d**) 76 °C; (**e**) 82 °C.

In addition complex modulus, the phase angle of bitumen samples also can be obtained via TS test, which reflects the hysteresis of strain to stress. Figure 2 presents the phase angle of bitumen samples before and after aging at 58 °C, 64 °C, 70 °C, 76 °C and 82 °C, respectively. It can be concluded from the figure that as the temperature rises, the phase angle of bitumen increases, but the increase is not obvious. The addition of plastic modifier reduces the phase angle of bitumen and the decrease becomes apparent with increasing plastic content. Similarly, the addition of SBR modifier further lowers the phase angle of the modified bitumen. This is achieved due to the addition of modifiers creating a support network in the modified bitumen, which leads to an enhanced elastic response for modified bitumen.

After long-term aging, the phase angle of the bitumen is substantially reduced. This is due to the content of polar components such as asphaltenes increasing as the bitumen ages and the elastic response of bitumen is higher after aging. This pattern also applies to plastic-modified bitumen and plastic/SBR composite-modified bitumen with different plastic content, indicating that the aging of modified bitumen results in an increase in elastic response of composite-modified bitumen.

### 3.3. Evaluation of Rutting Factor G*/sinδ

**Figure 3:**
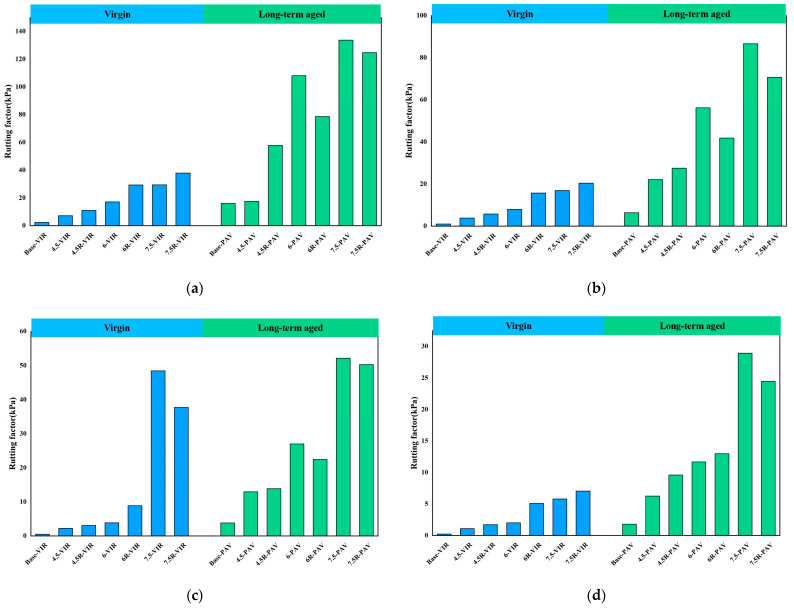

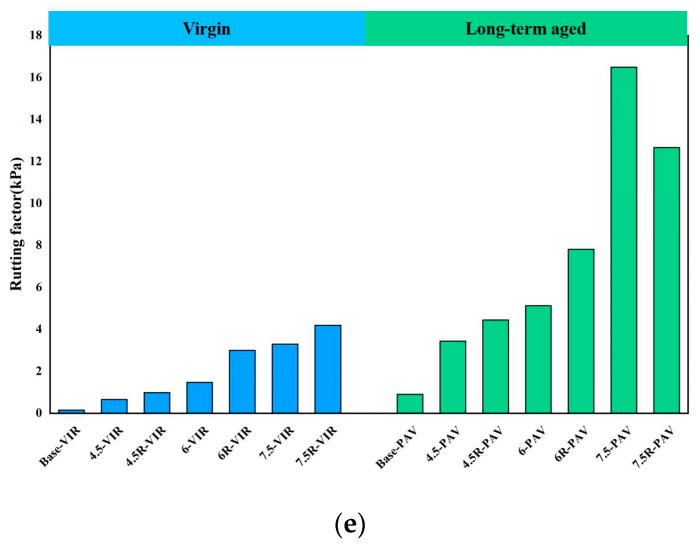
Rutting factor of bitumen before and after aging at: (**a**) 58 °C; (**b**) 64 °C; (**c**) 70 °C; (**d**) 76 °C; (**e**) 82 °C.

After obtaining the complex modulus and phase angel of bitumen, the rutting factor of bitumen was obtained, which is proven to correlate well with the high-temperature performance of bitumen. Figure 3 presents the rutting factor of bitumen before and after aging. As can be seen from the figure, the application of plastic improves the rutting factor of bitumen at different temperatures, and the increase in the rutting factor is more pronounced as the content of plastic increases, indicating the addition of plastic improves the high-temperature rutting resistance of bitumen. In addition, the addition of SBR does not lessen the improvement of the plastic on the high-temperature performance of the bitumen. On the contrary, the addition of SBR further improves the high-temperature performance of waste plastic-modified bitumen. After long-term aging, the rutting factor of bitumen increases obviously with the highest rutting factor of 7.5-PAV, indicating the temperature performance enhancement after bitumen long-term aging. It applies to all base bitumen, plastic-modified bitumen and plastic/SBR composite-modified bitumen.

### 3.4. Evaluation of Percent Recovery and Non-Recoverable Creep Compliance

**Figure 4:**
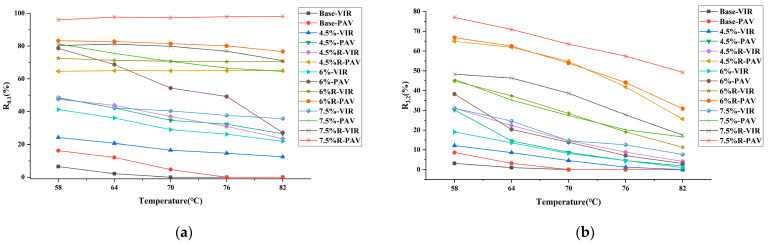
Percent recovery of bitumen sample: (**a**) R_0.1_; (**b**) R_3.2_.

The percent recovery of different bitumen samples at 0.1 and 3.2 kPa at five temperatures are measured via MSCR test, which characterizes the elastic deformation capacity of bitumen; results are shown in Figure 4. It can be concluded from the figure that for base bitumen, the percent recovery is low at both 0.1 and 3.2 kPa stress. Modification with plastic or SBR modifier leads to improved percent recovery of bitumen. In comparison, the modification of SBR plays a more vital role in improving the percent recovery of bitumen. Following the long-term aging process, the percent recovery of all base bitumen, plastic-modified bitumen and plastic/SBR composite-modified bitumen increases at five temperatures. For base bitumen, the recovery rate of bitumen after aging is still at a low level. For plastic-modified or plastic/SBR composite-modified bitumen, the improvement of recovery rate at 0.1 and 3.2 kPa after long-term aging is pronounced. Especially for 7.5R-PAV, the percent recovery of bitumen is close to 100%.

**Figure 5:**
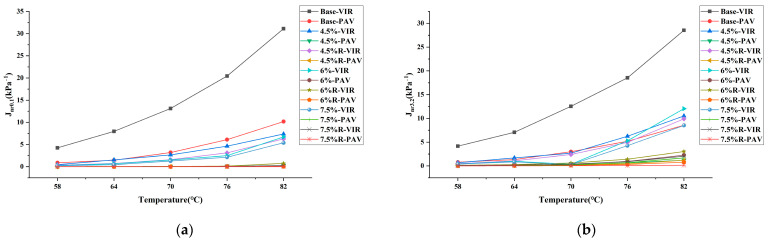
Non-recoverable creep compliance of bitumen sample: (**a**) R_0.1_; (**b**) R_3.2_.

Figure 5 shows the non-recoverable creep compliance of bitumen at 0.1 and 3.2 kPa. It is generally accepted that the non-recoverable creep compliance of bitumen correlates well with the resistance to rutting of modified bitumen at high temperature. The lower the non-recoverable creep compliance, the better the high-temperature performance of the bitumen. As concluded from the figure for base bitumen, the non-recoverable creep compliance values J_nr0.1_ and J_nr3.2_ are high, indicating the high-temperature performance of base bitumen is weak. In contrast, the addition of the plastic modifier reduces the non-recoverable creep compliance of bitumen. The decrease in non-recoverable creep compliance is evident as the amount of plastic modifier content increases. The additional incorporation of SBR further reduces the non-recoverable creep compliance of plastic-modified bitumen. Especially for 7.5% R-VIR, the non-recoverable creep compliance at 0.1 and 3.2 kPa is close to 0. As such, the higher plastic and SBR content is recommended to improve the high-temperature performance of bitumen. After long-term aging, the J_nr0.1_ and J_nr3.2_ of all base bitumen, plastic-modified bitumen and plastic/SBR composite-modified bitumen decreases dramatically. Especially for plastic-modified bitumen and plastic/SBR composite-modified bitumen, the J_nr0.1_ and J_nr3.2_ of bitumen after aging are close to 0. As such, for modified bitumen, the high-temperature rutting resistance of bitumen after long-term aging is not a concern. To improve the performance of plastic-modified bitumen, existing studies have tried adding recycled crumb rubber to plastic bitumen. The reported findings in their studies suggested the incorporation of 11% content crumb rubber to 6% plastic-modified bitumen increased non-recoverable creep compliance at 3.2 kPa of bitumen at 58 °C by 20% [30]. The high-temperature performance of bitumen decreased after composite modification with crumb rubber and plastic. For comparison, the incorporation of 3% SBR to 6% plastic-modified bitumen reduced non-recoverable creep compliance at 3.2 kPa of bitumen at 58 °C by 96.3%. The above results show that the composited modification of SBR is more beneficial for the high-temperature performance of bitumen compared to crumb rubber.

### 3.5. Evaluation of Molecular Weight Distribution

**Figure 6:**
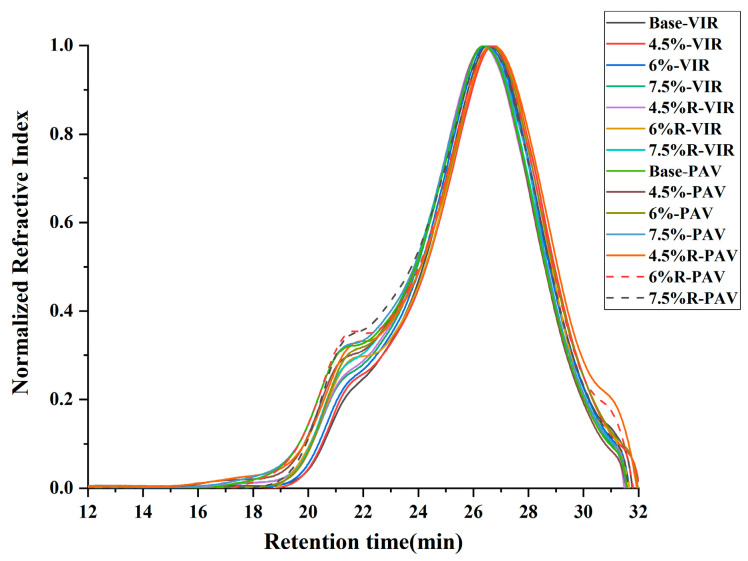
Molecular weight distribution chromatograms of bitumen before and after aging.

Figure 6 shows the molecular weight distribution chromatograms of bitumen before and after aging. As can be seen from the figure for bitumen samples, there is a rising peak in the chromatogram around 21 to 22 min (corresponding to molecular weights of about 17,800 to 11,000 Daltons). The elevation of the peak at 21 to 22 min is higher in the chromatogram of the modified bitumen compared to the base bitumen. This indicates that a molecular aggregation process takes place during the modification process. The ongoing aggregation of small molecules into large molecules leads to an increase in the content of large molecular weight substances. After long-term aging, the higher peaks at 21 to 22 min for different bitumen chromatograms are found, suggesting the small molecule aggregation process also occurs during the long-term aging of bitumen.

**Figure 7:**
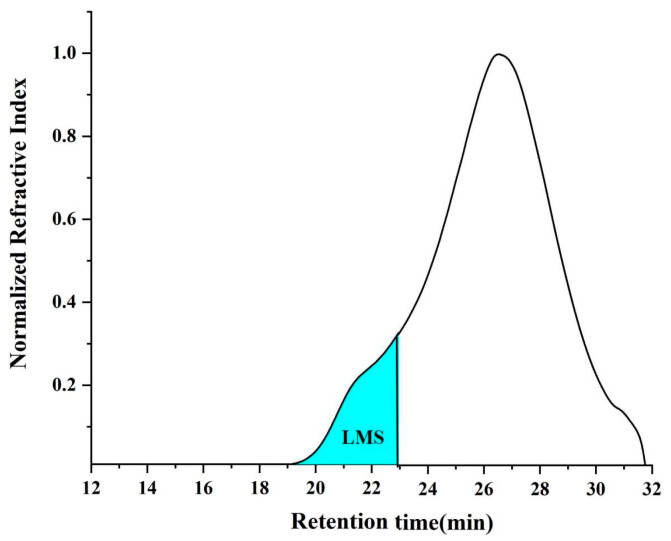
Schematic diagram of the division.

To quantify the bitumen chromatogram, Li et al., [31,32] divided the bitumen chromatogram into large, medium and small molecules based on molecular weight. A schematic diagram of the division is shown in Figure 7. Using this chromatogram division method, the large molecule size (LMS) percentage of bitumen can be calculated from the following Equation (3).

(3) $$LMSP=\frac{Area_{LMS}}{\mathrm{Total}\;\mathrm{area}\;\mathrm{under}\;\mathrm{chromatogram}}$$

where *LMSP* represents the large molecule size percentage in bitumen, %.

**Figure 8:**
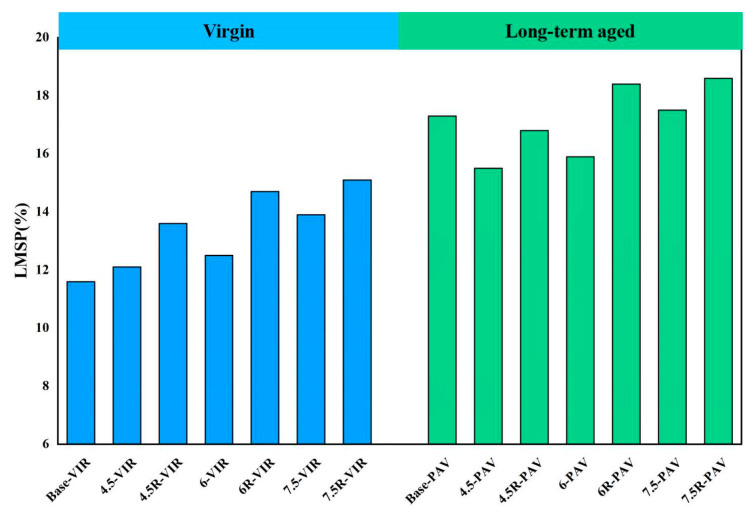
LMSP of bitumen before and after long-term aging.

The corresponding *LMSP* of base bitumen, plastic-modified bitumen and plastic/SBR composite-modified bitumen before and after long-term aging is calculated via Equation (3); results are shown in Figure 8. It is quite clear from the figure that the *LMSP* of bitumen increases after the modification by plastic and SBR. The addition of 4.5%, 6% and 7.5% plastic modifier to the base bitumen increases the *LMSP* by 4.3%, 7.8% and 19.8%. In addition, the addition of SBR to 4.5-VIR, 6-VIR and 7.5-VIR increases the *LMSP* by 12.4%, 17.6% and 8.6%, respectively. In addition, the *LMSP* of bitumen also increases with long-term aging. This indicates that the aggregation of small molecules occurs during the both modification and aging process of bitumen.

**Figure 9:**
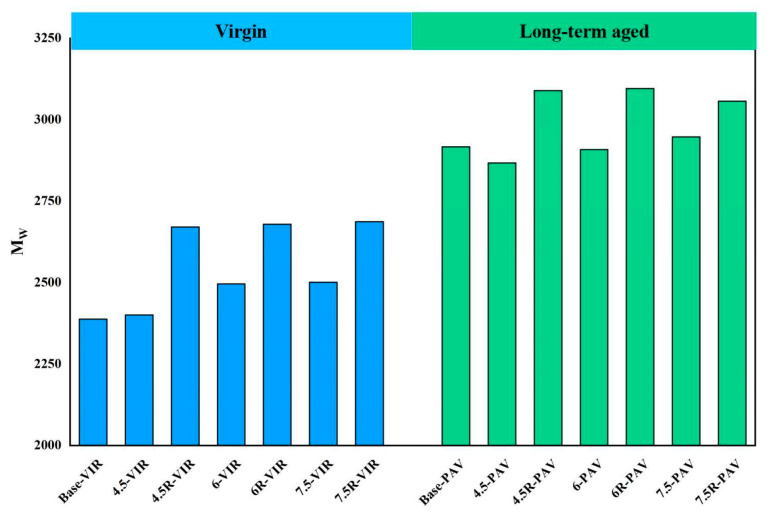
*M_w_* of bitumen before and after long-term aging.

In addition to the LMSP of bitumen, the GPC test also gives the weight average molecular weight ($\bar{M_{W}}$) of the sample, which is derived via the following equation. $\bar{M_{W}}$ is currently believed to reflect changes in large molecule size in the bitumen samples. Figure 9 presents the weight average molecular weight of base bitumen, plastic and plastic/SBR composite-modified bitumen before and after aging. As can be seen from the figure, the $\bar{M_{W}}$ of bitumen slightly increases with the modification by plastic. The addition of 4.5%, 6% and 7.5% plastic modifier to the base bitumen increases the $\bar{M_{W}}$ by 0.5%, 4.5% and 4.7%, respectively. However, the lifting effect is not as effective as that with SBR modification. The addition of SBR to the 4.5-VIR, 6-VIR and 7.5-VIR increases the $\bar{M_{W}}$ by 11.8%, 7.4% and 7.5%,respectively. In addition, the $\bar{M_{W}}$ of bitumen increases after a long-term aging process, which is due to the aggregation of small molecules after aging.

(4) $$\bar{M_{W}}=\sum_{i=1}^{n}\frac{w_{i}\times M_{i}}{w_{i}}$$

where, *M_i_* represents the molecular weight; *w_i_* represents the weight of molar mass *M_i_*.

To evaluate the aging resistance of bitumen in terms of the change in molecular size, two evaluation indexes, the large molecular size aging index (*LAI*) and the molecular weight aging index (*MAI*) (Equations (5) and (6)), are proposed. Table 5 presents the *LAI* and *MAI* of base bitumen, plastic-modified bitumen and plastic/SBR composite-modified bitumen. It can be concluded from the table that the base bitumen has the highest *LAI* and *MAI*. For *LAI*, the addition of 4.5%, 6% and 7.5% plastic modifier to the base bitumen decreases *LAI* by 14.1%, 14.8% and 14.8%, respectively. For *MAI*, the addition of 4.5%, 6% and 7.5% plastic modifier to the base bitumen decreases *MAI* by 2.5%, 4.1% and 3.3%, respectively, indicating that the base bitumen is less resistant to aging. The *LAI* and *MAI* of plastic-modified bitumen and plastic/SBR composite-modified bitumen are lower, indicating that the addition of modifiers improves the aging resistance of the bitumen.

(5) $$LAI=\frac{LMSP_{age}}{LMSP_{vir}}$$

(6) $$MAI=\frac{\bar{M_{W\mathrm{age}}}}{\bar{M_{Wvir}}}$$

where *LAI*, *MAI* are the evaluation indexes; *LMSP_vir_* is the *LMSP* of bitumen before aging,%; *LMSP_age_* is the *LMSP* of bitumen after aging,%.$\bar{M_{Wvir}}$ is the weight average molecular weight of bitumen before aging; $\bar{M_{W\mathrm{age}}}$ is the weight average molecular weight of bitumen after aging;

### 3.6. Correlation between Molecular Weight and Rheological Properties

To analyze the effect of molecular weight on the rheological properties of plastics and plastic/SBR-modified bitumen, correlations between *LMSP*, $\bar{M_{W}}$ and rutting factor, J_nr3.2_ at 58 °C of plastic and plastic/SBR-modified bitumen were established using Pearson correlation analysis. Table 6 shows the correlation between *LMSP*, $\bar{M_{W}}$ and rheological properties of bitumen. As can be seen from the table, the $\bar{M_{W}}$ and rheological properties of plastic and plastic/SBR-modified bitumen do not show a good linear correlation. This is due to the use of a 0.45-micron filter to filter the dissolved bitumen sample prior to the experiment. The size of the plastic particles is greater than 0.45-micron, so the plastic phase will still remain on the filter after filtration. Thus, the $\bar{M_{W}}$ measured via GPC does not provide a realistic representation of the $\bar{M_{W}}$ of the plastic-modified bitumen. In contrast, the *LMSP* captures the molecular weight distribution of bitumen, rather than the actual molecular weight. Thus, the *LMSP* exhibits a better correlation with the rheological properties of modified bitumen. This indicates that the molecular weight distribution of bitumen affects its rheological properties to some extent.

## 4. Conclusions

With the aid of DSR and GPC tests, the rheological properties and molecular weight distribution of base bitumen, plastic, and plastic/SBR-modified bitumen before and after long-term aging were measured. According to the measured results, the following conclusions were summarized:

1. Modification by plastic improves the high-temperature performance of bitumen. It increases complex modulus, rutting factor and percent recovery of bitumen while it decreases the phase angle and non-recoverable creep compliance of bitumen. The integrate modification of plastic and SBR generates an elastic supporting network of bitumen, further enhancing the high-temperature performance of modified bitumen. The enhancement effect is more pronounced with increasing plastic inclusion.
2. The aggregation of small molecules occurs during the modification by plastic and SBR, as the large size molecule percentage in bitumen increases. During the long-term aging process, the small molecules in the bitumen also undergo aggregation reactions, and the LMSP and $\bar{M_{W}}$ of bitumen increases.
3. For all three plastic-modified bitumen, the CAI, *LAI* and *MAI* are smaller than those of base bitumen. The aging resistance of bitumen is improved by an average of about 10% after the addition of plastic modifiers, and it improves more significantly with increasing plastic content. After the addition of SBR modifier, the aging resistance of modified bitumen is further improves.

The $\bar{M_{W}}$ and rheological properties of plastic and plastic/SBR-modified bitumen do not correlate well, with correlation coefficients less than 0.6. In contrast, the *LMSP* of bitumen exhibits a better correlation with rheological properties of the modified bitumen, with correlation coefficients exceeding 0.7. The molecular weight distribution of bitumen affects its rheological properties to some extent.

It is recommended to use SBR for composite modification of plastic-modified bitumen, which improves the high-temperature performance and aging resistance of modified bitumen. However, only one plastic modifier was utilized in this study. Future study is envisaged to verify the applicability of this rule using more types of plastic modifiers.

## Figures and Tables

**Table 1:** Abbreviations used in this studies.

Abbreviations	Full Description
SBR	Styrene butadiene rubber
SBS	Styrene-butadiene-styrene
GPC	Gel permeation chromatography
PAV	Pressure aging vessel
MSCR	Multiple stress creep recovery
TS	Temperature sweep
LMSP	Large molecule size percentage
CAI	Complex modulus aging index
LAI	Large molecular size distribution aging index
MAI	Molecular weight aging index

**Table 2:** Basic properties of bitumen.

Properties	Results	Test Method
Penetration (25 °C, 100 g, 5 s, 0.1 mm)	67.8	ASTM D5
Softening point (°C)	48.3	ASTM D36
Ductility (15 °C, 5 cm/min, cm)	>100	ASTM D113
Rotary viscosity (135 °C, Pa·s)	0.41	ASTM D4402

**Table 3:** Detailed preparation process of bitumen used in this study and their identifications.

Bitumen	Waste Plastic Modifier	SBR	Long-Term Aging	Identifications
Base bitumen	-	-	-	Base-VIR
	-	-	20 h PAV	Base-PAV
	4.5%	-	-	4.5-VIR
	4.5%	-	20 h PAV	4.5-PAV
	4.5%	3%	-	4.5R-VIR
	4.5%	3%	20 h PAV	4.5R-PAV
	6%	-	-	6-VIR
	6%	-	20 h PAV	6-PAV
	6%	3%	-	6R-VIR
	6%	3%	20 h PAV	6R-PAV
	7.5%	-	-	7.5-VIR
	7.5%	-	20 h PAV	7.5-PAV
	7.5%	3%	-	7.5R-VIR
	7.5%	3%	20 h PAV	7.5R-PAV

**Table 4:** CAI of different bitumen.

Temperature	58 °C CAI	64 °C CAI	70 °C CAI	76 °C CAI	82 °C CAI
Base bitumen	6.82	6.16	7.39	7.51	5.87
Base bitumen + 4.5% plastic	5.96	5.43	5.48	5.42	4.98
Base bitumen + 4.5% plastic + 3% SBR	4.92	4.47	4.19	5.16	4.15
Base bitumen + 6% plastic	5.68	6.42	6.36	5.38	3.37
Base bitumen + 6% plastic + 3% SBR	2.58	2.57	2.44	2.46	2.51
Base bitumen + 7.5% plastic	4.21	4.75	4.98	4.64	4.67
Base bitumen + 7.5% plastic + 3% SBR	2.75	2.75	3.19	2.43	2.01

**Table 5:** LAI and MAI of different bitumen.

Bitumen	*LAI*	*MAI*
Base bitumen	1.49	1.22
Base bitumen + 4.5% plastic	1.28	1.19
Base bitumen + 4.5% plastic + 3% SBR	1.24	1.16
Base bitumen + 6% plastic	1.27	1.17
Base bitumen + 6% plastic + 3% SBR	1.25	1.16
Base bitumen + 7.5% plastic	1.27	1.18
Base bitumen + 7.5% plastic + 3% SBR	1.23	1.14

**Table 6:** Correlation between molecular weight and rheological properties.

Bitumen Type	Regression Equation	R^2^
Plastic-modified bitumen	$G^{*}/\sin\delta =-264.71+21.75LMSP$	0.71
	$J_{nr3.2}=1.68-0.09LMSP$	0.74
	$G^{*}/\sin\delta =-402.69+0.17\bar{M_{W}}$	0.48
	$J_{nr3.2}=2.35-7.43\times 10^{-4}\bar{M_{w}}$	0.62
Plastic/SBR-modified bitumen	$G^{*}/\sin\delta =-243.76+18.54LMSP$	0.84
	$J_{nr3.2}=0.16-0.01LMSP$	0.75
	$G^{*}/\sin\delta =-366.45+0.15\bar{M_{W}}$	0.53
	$J_{nr3.2}=0.21-6.03\times 10^{-5}\bar{M_{w}}$	0.37

## Data Availability

The data presented in this study are available on request from the corresponding author.
